# *RUBY*-Mediated Visual Selection Facilitates Transgenic Screening and Red Floral Pigmentation in *Petunia* × *hybrida*

**DOI:** 10.3390/plants15060886

**Published:** 2026-03-12

**Authors:** Jian Yao, Fanzhuang Yan, Ajithan Chandrasekaran, Theint Theint Aung, Sangrim Youn, Youngtak Kim, Geung-Joo Lee

**Affiliations:** 1 Department of Smart Agriculture Systems, Chungnam National University, Daejeon 34134, Republic of Korea; yaojiansdau@163.com (J.Y.); yanfanzhuang@126.com (F.Y.); knase1894@naver.com (Y.K.); 2 Department of Horticulture, Chungnam National University, Daejeon 34134, Republic of Korea; ajithancbiotech@gmail.com (A.C.); theinttheintaung508@gmail.com (T.T.A.); rimmyy0826@naver.com (S.Y.)

**Keywords:** *Agrobacterium*-mediated transformation, *Petunia* **×** *hybrida*, *RUBY* reporter gene, floral development

## Abstract

Visual transgenic marker systems enabling rapid and non-destructive transformant detection are crucial for efficient plant genetic transformation and selection. *RUBY* is a highly effective reporter system based on betalain biosynthesis; however, its application to species outside of the order Caryophyllales (i.e., species lacking betalains) has not been established. In this study, we performed the first systematic evaluation of the *RUBY* system using *Petunia* × *hybrida* lines obtained via *Agrobacterium*-mediated gene transformation. Stable *RUBY* transgenic plants were obtained from an optimized transformation and organogenesis system. The transgenic lines displayed a gradient of betalain accumulation, with pigment intensity positively correlated with *RUBY* expression levels and metabolite contents. In a morphological analysis, there was a negative correlation between *RUBY* expression and corolla opening, suggesting that *RUBY* pigment overaccumulation is associated with altered floral development and morphology. *RUBY* overexpression significantly reduced expression levels of gibberellin biosynthetic genes (*PhGA20ox1* and *PhGA3ox1*) and flowering- and senescence-related regulators (*PhNF-YC2* and *PhOBF1*). These findings indicate that high-level betalain accumulation is associated with changes in floral development and gene expression, highlighting both the utility of the *RUBY* system as a visual reporter and the importance of carefully evaluating potential developmental effects under strong expression conditions.

## 1. Introduction

Plant genetic transformation and genome editing technologies, such as the CRISPR-Cas9 system, have revolutionized agricultural research by enabling precise genome modifications [1,2]. *Agrobacterium tumefaciens*-mediated transformation allows the stable integration of editing constructs into plant genomes, generating transgenic lines that often require the subsequent elimination of transgenic components to obtain non-transgenic edited plants for commercialization [3]. Efficient identification of positive transformants is critical for successful plant transformation. Although various visible marker systems, such as Green Fluorescent Protein (GFP), Red Fluorescent Protein (RFP), mCherry, and YFP, have been developed for rapid screening at early transformation stages, their dependence on specialized light sources or imaging equipment limits their practicality in routine screening [4,5]. The classical β-glucuronidase (*GUS*) reporter gene [6], while utilized extensively for gene expression analyses, requires invasive detection methods involving tissue sacrifice and substrate application, increasing contamination risks and reducing screening efficiency.

To address the limitations of conventional marker genes, the *RUBY* reporter system has been developed based on betalain biosynthesis. Betalains, including betacyanins and yellow betaxanthins, are naturally occurring nitrogen-containing plant pigments that produce diverse colors. The betalain biosynthetic pathway involves the conversion of tyrosine to l-DOPA by cytochrome P450 enzymes (CYP76AD1 or CYP76AD6), followed by the formation of betalamic acid via DOPA 4,5-dioxygenase (DOD). Betalamic acid then conjugates with amino acids to form yellow betaxanthins or reacts with cyclo-DOPA to produce betanidin, the precursor for red-violet betacyanins [7]. The *RUBY* construct integrates CYP76AD1, DODA, and a glucosyltransferase into a single open reading frame linked by 2A peptides and driven by the 35S promoter. This compact design minimizes plasmid size while ensuring coordinated expression of all pathway enzymes. The *RUBY* system enables non-invasive, continuous, and cost-effective visualization of plant cell activity without the need for specialized instruments or destructive assays [8].

Betalains are uniquely confined to the order Caryophyllales, where they replace anthocyanins functionally [9]. Beyond providing vivid coloration, these secondary metabolites possess strong antioxidant activity and are implicated in cellular signaling and stress responses [10,11]. By efficiently scavenging reactive oxygen species, betalains help maintain redox balance and enhance tolerance to abiotic stresses, such as drought, heat, and salinity [12]. They also inhibit indole-3-acetic acid oxidase, suggesting a link between betalain metabolism and auxin-mediated growth regulation [13]. Nevertheless, betalain accumulation may entail physiological costs. Extracts from red beet (*Beta vulgaris*), a betalain-rich species, exhibit allelopathic effects, although the compounds responsible have not been determined [14]. Moreover, betalain biosynthesis requires substantial metabolic investment, consuming tyrosine and energy, which may perturb cellular homeostasis when heterologously expressed, as in the *RUBY* system [11]. However, the physiological effects of betalain overaccumulation in non-Caryophyllales species remain poorly understood. Systematic analyses of effects of heterologous betalain biosynthesis on plant growth, hormone signaling, and development are lacking.

*Petunia* × *hybrida*, a model for floral pigmentation and morphogenesis [15], provides an ideal system to evaluate the effects of betalain and the efficacy of the *RUBY* system. Here, we generated *RUBY*-expressing petunia lines via *Agrobacterium*-mediated transformation to assess reporter performance and to examine how ectopic betalain accumulation influences floral development. Our findings show that betalain biosynthesis modulates gibberellin metabolism and transcriptional regulation, revealing a dual role of *RUBY* as both a visible marker and a developmental regulator.

## 2. Results

### 2.1. A High BAP Concentration Optimizes 35S:RUBY Petunia Regeneration

**Figure 1:**
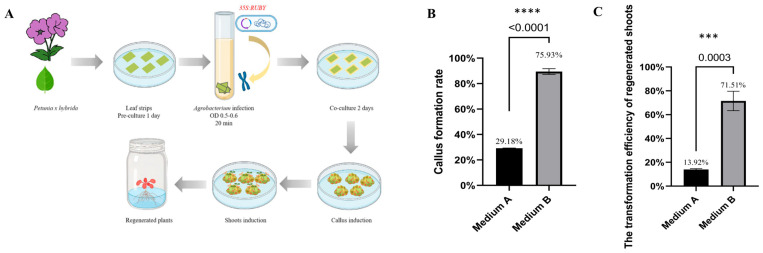
Identification of optimal regeneration medium in *Petunia* × *hybrida* using *RUBY* reporter gene. (**A**) Schematic representation of *Agrobacterium*-mediated transformation of *Petunia* × *hybrida* via the leaf disc method. Leaf strips were pre-cultured for 1 day before being infected with *Agrobacterium* tumefaciens (OD_600_ = 0.5–0.6) carrying the *35S:RUBY* construct for 20 min. After 2 days of co-cultivation, explants were transferred to callus induction, shoot induction, and regeneration media sequentially to obtain transgenic plants. (**B**) Callus formation rate of explants cultured on Medium A and Medium B. (**C**) Transformation efficiency of regenerated shoots on Medium A and Medium B. Data are presented as mean ± SD (*n* = 3 independent plates). Statistical analysis was performed using an unpaired two-tailed Student’s *t*-test. Statistical significance was defined as **** (*p* < 0.0001) and *** (*p* < 0.001).

Explants infected with *35S:RUBY* were cultured on two different regeneration media, Medium A and Medium B. Within one week, visible callus formation appeared on the explants, accompanied by red pigmentation in callus tissues, indicating successful transformation. By the fourth week, callus proliferation increased markedly, and red coloration became more intense in the transformed callus (Figure 1). The callus induction rate on Medium B was 75.93%, which was significantly higher than the estimate of 29.18% on Medium A (Figure 1B). Approximately 5 weeks post-infection, the red callus became more prominent, and shoot emergence began. By the sixth week, numerous shoots developed, with red shoots accounting for 71.51% of total shoots on Medium B, compared with only 13.92% on Medium A (Figure 1C). These results demonstrate that Medium B, containing a higher BAP concentration (2 mg/L BAP), enhanced both callus regeneration and transformation efficiency in petunia.

### 2.2. Optimization of Pre-Culture and Infection Durations for Efficient 35S:RUBY Petunia Transformation

**Figure 2:**
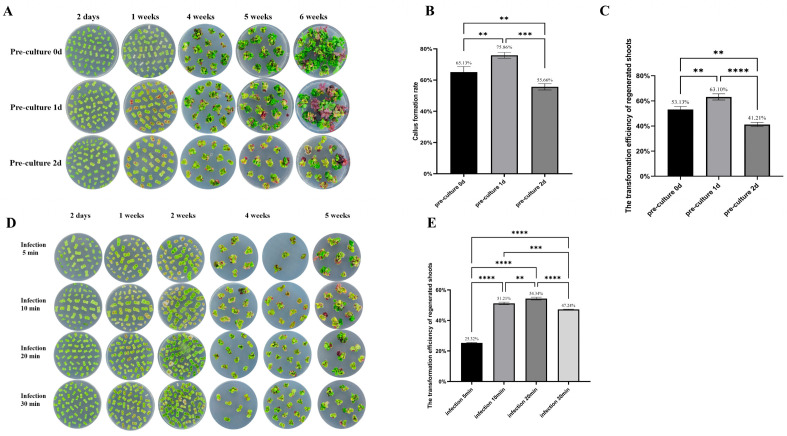
*RUBY* reporter guided optimization of *Agrobacterium* infection in *Petunia* × *hybrida*. (**A**) Morphological observation of explants after *Agrobacterium* infection and cultivation at 2 days, 1 week, 4 weeks, 5 weeks, and 6 weeks under different pre-culture durations (0, 1, and 2 days). (**B**) Callus formation rates of explants subjected to different pre-culture durations. (**C**) Transformation efficiency of regenerated shoots under various pre-culture conditions. (**D**) Representative images of callus and shoot regeneration after *Agrobacterium* infection for 5, 10, 20, or 30 min, observed at different time points (2 days, 1–5 weeks post-infection). (**E**) Transformation efficiency of regenerated shoots under different infection durations. Data represent means ± SD (*n* = 3). Statistical significance was determined by one-way ANOVA with Tukey’s post hoc test. Statistical significance was defined as **** (*p* < 0.0001), *** (*p* < 0.0002), ** (*p* < 0.001) and * (*p* < 0.01).

Pre-culture is considered essential for successful transformation in many plant species, typically involving a 2-day incubation period. In petunia, transformation was achieved even without pre-culture (Figure 2A). When freshly excised explants were directly infected with *Agrobacterium*, red coloration—likely indicative of transient *RUBY* expression—appeared within one week. These explants exhibited rapid growth, with 65.13% (Figure 2B) forming callus tissue; however, only 53.13% (Figure 2C) developed red shoots after 6 weeks. One day of pre-culture resulted in the highest rates of callus formation and stable transformation. A 2-day pre-culture led to slower growth, higher contamination, and the lowest transformation efficiency. These findings suggest that a one-day pre-culture period is optimal for petunia transformation using the *RUBY* marker.

The duration of explant immersion in the *Agrobacterium* suspension, referred to as the infection time, plays a crucial role in determining transformation efficiency. While extended infection times can increase the number of infected cells and potentially enhance transformation efficiency, they also raise the risk of *Agrobacterium* overgrowth and tissue necrosis. Thus, identifying the shortest infection period that maintains high efficiency is crucial to minimize contamination and improve reproducibility and operational efficiency. We evaluated four infection durations: 5, 10, 20, and 30 min (Figure 2D). After 6 weeks of culture, explants infected for 5 min exhibited a transformation rate of only 25.32%. Both 10 and 20 min treatments improved the transformation efficiency significantly, with rates exceeding 50%; A duration of 20 min was the most effective, achieving a transformation rate of 54.34%. Explants infected for 30 min showed extensive *Agrobacterium* overgrowth during subsequent culture, leading to a reduced transformation efficiency of 47.24% (Figure 2E). Collectively, these results indicate that a 20 min infection time is optimal for petunia transformation using the *RUBY* marker.

### 2.3. Molecular Characterization and Phenotypic Analysis of RUBY Transgenic Petunia

**Figure 3:**
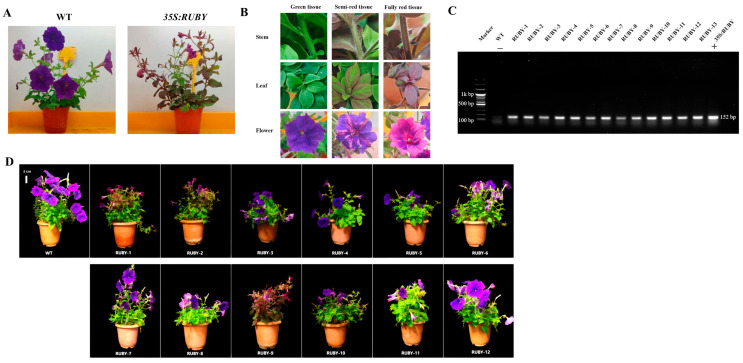
Phenotypic and molecular characterization of *35S:RUBY* transgenic petunia lines. (**A**) Representative phenotypes of WT and independent *35S:RUBY* transgenic petunia lines. (**B**) Chimeric tissue characteristics observed in *35S:RUBY* transgenic plants. Due to differential betalain accumulation, three distinct tissue types are present: green tissue, semi-red tissue, and fully red tissue. Images show stem, leaf, and flower pigmentation in each tissue type. (**C**) PCR validation of transgene insertion in transgenic lines using gene-specific primers. M: DNA marker; WT: wild type (negative control “−”); “+” indicates positive control (*35S:RUBY* plasmid DNA); 152 bp: expected amplicon size. (**D**) Phenotypes of *35S:RUBY* transgenic petunia lines at the age of 2 months. All *RUBY*-1-12 transgenic plants exhibited pigment deposition to varying extents. Among them, *RUBY*-1, *RUBY*-2, and *RUBY*-9 showed relatively prominent accumulation of red betanin. The plants were eight weeks old at the time of observation, and the scale bar corresponds to 5 cm.

Using the optimized transformation protocol described above, we obtained 12 acclimated (T_0_) *35S:RUBY* transgenic plants (Figure 3A,D). The transgenic plants exhibited three distinct tissue types: fully green, semi-red, and fully red sectors (Figure 3B). Genomic DNA was extracted, and PCR using *35S:RUBY* specific primers successfully amplified a 152 bp fragment in all transgenic lines but not in the wild-type (WT) controls (Figure 3C), confirming the stable integration of the *RUBY* gene.

**Figure 4:**
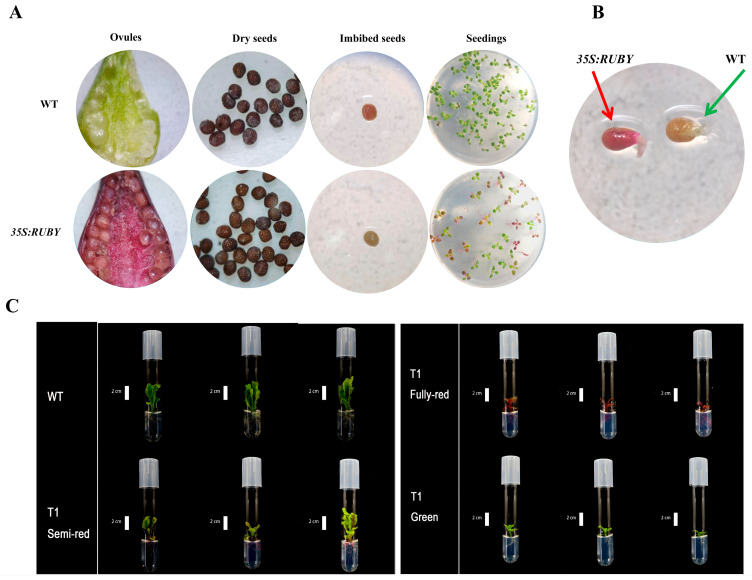
Seed development and germination phenotypes in wild-type (WT) and transgenic *RUBY* plants. (**A**) Comparison of ovule morphology, dry seeds, imbibed seeds, and germinating seedlings between WT and transgenic *RUBY* plants. WT ovules and seeds appear yellowish, whereas transgenic *RUBY* lines exhibit a distinct red pigmentation due to *RUBY* expression. (**B**) Identification of transgenic seeds by visible pigmentation. The red-colored seed (indicated by the red arrow) expresses the *RUBY* reporter gene under the 35S promoter (*35S:RUBY*), while the non-pigmented seed (green arrow) represents the non-transgenic control. (**C**) Phenotypic variation in regenerated T1 plants and its statistical distribution. The seeds are from *RUBY*-1 T0 plant. WT plants exhibit no pigmentation. T1 lines display varying degrees of red coloration due to *RUBY* expression, including green, semi-red, and fully red phenotypes. The plants were three weeks old at the time of observation, and the scale bar corresponds to 2 cm.

Phenotypically, *RUBY* transgenic plants showed normal vegetative growth and reproductive capacity compared with WT, flowering and producing viable seeds. However, in some high betalain accumulation lines, reduced petal expansion and incomplete flower opening were observed. An anatomical analysis revealed that the ovaries and ovules of WT plants were pale green, whereas those of *35S:RUBY* transgenic plants were red (Figure 4A). Mature dry seeds appeared dark brown in both genotypes, making visual distinction difficult. However, following imbibition, the red seed coat of transgenic seeds became clearly visible under a microscope. Both transgenic and WT seeds germinated normally. After 2 days, hypocotyls began to elongate, and red pigment first accumulated in the hypocotyls (Figure 4B). In T_1_ seedlings, aerial tissues initially appeared green, indicating delayed *RUBY* expression, while red coloration was already visible in the root systems, making root pigmentation a more reliable marker for identifying transgenic individuals.

A segregation analysis of T_1_ progeny from three representative independent lines (*RUBY*-1, -2, and -9) showed distinct phenotypic ratios (Table 1). For line #1, 31.25% of surviving seedlings were fully green, 62.50% semi-red, and 6.25% fully red, with a survival rate of 90.91%. Line #2 exhibited a higher proportion of fully red seedlings (25%) but also the highest mortality rate (29.82%), resulting in a survival rate of 70.18%. Line #3 displayed intermediate values, with 25.45% fully green, 53.64% semi-red, and 20.91% fully red seedlings and a survival rate of 84.62%. These results indicate that *RUBY*-based pigmentation markers can facilitate early-stage transgenic identification in petunia, although variation exists among different transformation events.

### 2.4. RUBY Expression Is Positively Correlated with the Betalain Content

**Figure 5:**
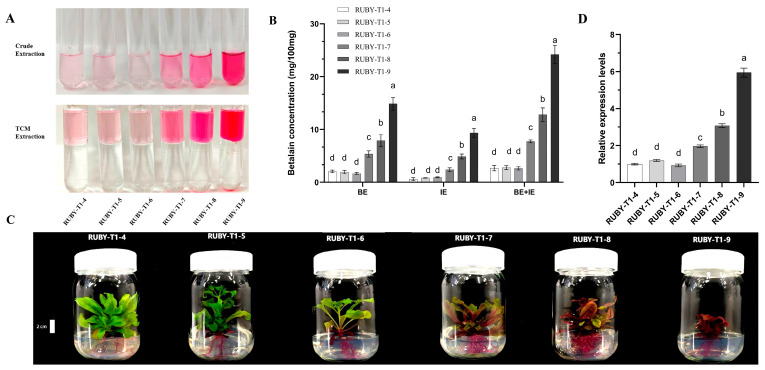
Betalain extraction and *RUBY* gene expression in transgenic petunia lines. (**A**) Representative images of betalain pigment extracted from different *35S:RUBY* transgenic lines (*RUBY*-T1-4, -5, -6, -7, -8, and -9) using two different extraction methods: Crude Extraction (upper panel) and TCM Extraction (lower panel). (**B**) Quantification of betalain concentration in different tissue types: BE (basal explant), IE (intermediate explant), and BE+IE (mixed). Different letters above bars indicate statistically significant differences (*p* < 0.05). (**C**) Phenotypes of transgenic lines grown in tissue culture jars showing variation in pigmentation intensity. The plants were five weeks old at the time of observation, and the scale bar corresponds to 2 cm. (**D**) qRT-PCR analysis of *RUBY* expression levels in different transgenic lines. Three clonal plants per transgenic line were used as biological replicates. Data are presented as mean ± SD (*n* = 3). Statistical differences were analyzed by one-way ANOVA followed by Tukey’s test (*p* < 0.05).

Betalains were extracted from transgenic leaves using methanol at various concentrations, purified using trichloromethane, and quantified spectrophotometrically. Betacyanins (red) and betaxanthins (yellow) are characteristic pigments in Caryophyllales species but are absent in *Solanaceae*, such as petunia. WT leaves showed no absorbance at 532 nm, confirming the absence of betacyanins; however, background absorbance at 482 nm was detected, likely from flavonoids. In *RUBY* transgenics, betalains were efficiently extracted using 0–60% methanol, with 40% methanol yielding the maximal betaxanthin concentration and minimal background interference (Supplementary Figure S1). Therefore, 40% methanol is recommended as the optimal concentration for efficient betalain extraction and pigment separation. Six transgenic T_1_ (seeds from *RUBY*-1) lines (*RUBY*-T1-4, -5, -6, -7, -8, and -9) were analyzed, and varying intensities of red pigmentation were observed (Figure 5C). A visual assessment revealed that plants with darker red coloration accumulated more betalains. Specifically, lines *RUBY*-T1-4, -5, and -6 displayed lighter pigmentation and lower betalain contents, whereas *RUBY*-T1-9 (Figure 5A,B), which exhibited the deepest red coloration, showed the highest betalain concentrations. A quantitative reverse transcription PCR (qRT-PCR) analysis confirmed that *RUBY* transcript levels were highest in *RUBY*-T1-9 (Figure 5D). These results indicate clear positive correlations among *RUBY* expression levels, betalain contents, and visible pigmentation.

### 2.5. Ectopic RUBY Expression Reduces Petal Expansion and Perturbs Flower Opening in Petunia

**Figure 6:**
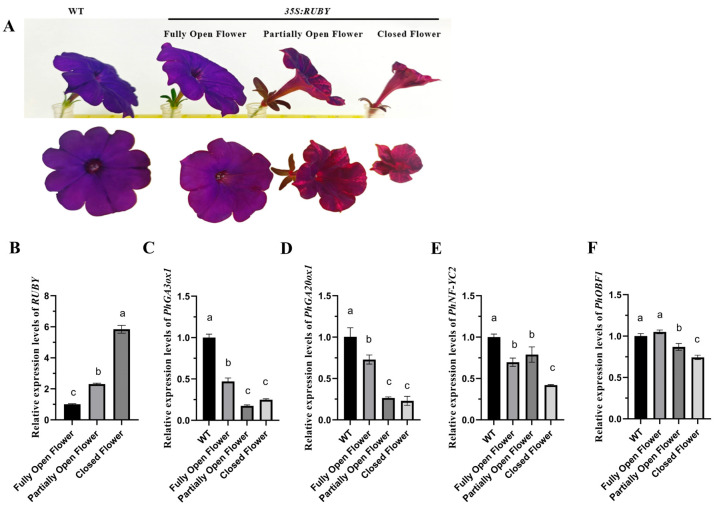
Effects of *RUBY* on the expression of flowering-related genes. (**A**) Flower phenotype of *35S:RUBY* transgenic petunia and WT: closed flower, partially open flower, and fully open flower. Top row: side view; bottom row: front view. Increased pigmentation is observed as flower opening progresses. (**B**–**F**) Relative expression levels of *PhGA20ox1*, *PhGA3ox1*, *PhOBF1*, *PhNF-YC2*, and *RUBY* in petals of different *35S:RUBY* transgenic petunia. Data are presented as mean ± SD (*n* = 4 independent lines). Three biological replicates per line were averaged prior to one-way ANOVA with Tukey’s multiple comparison test (*p* < 0.05). Different letters indicate statistically significant differences. Closed flowers are from *RUBY*-1, 2, 3, 9; partially open flowers are from *RUBY*-4, 5, 8, 10; and fully open flowers are from *RUBY*-6, 7, 11, 12 (Figure 3D).

Both *RUBY* transgenic (*RUBY*-1-12 lines in T_0_) and WT petunias grew normally, flowered, and, despite some abnormalities in transgenic flower opening, produced fertile seeds. WT flowers exhibited a deep purple color and wide corolla opening at full bloom, allowing clear visualization of floral organs. *RUBY* transgenic flowers exhibited three distinct pigmentation patterns—purple-red stripes, fully red petals, or fully purple petals. These three patterns correspond to three flower opening states: closed, partially open, and fully open flowers (Figure 6A). Furthermore, all three petal types could be observed simultaneously within the same partially pigmented transgenic plant. A quantitative analysis of flower phenotypes (Table 2) revealed that WT plants exclusively produced fully open, fully purple flowers (18 out of 18 total petals). In contrast, several *RUBY* lines showed a significant shift towards closed or partially open flowers. For instance, *RUBY*-9 exhibited a high proportion of closed, fully red flowers (14 out of 16 total petals), resulting in a significantly reduced mean petal diameter of 3.23 ± 0.79 cm compared with that of WT plants (5.24 ± 0.77 cm, *p* < 0.001). Other lines, such as *RUBY*-1 (mean petal diameter 3.80 ± 1.02 cm, *p* < 0.001) and *RUBY*-2 (mean petal diameter 3.61 ± 1.04 cm, *p* < 0.001), also displayed significantly smaller petal diameters than those of WT plants. These observations suggest a negative correlation between the extent of red betanin coloration and the degree of corolla opening.

To further determine whether reduced corolla opening is associated with gibberellin limitation, exogenous GA_3_ application was performed on high-*RUBY* lines. However, GA_3_ treatment did not significantly restore petal expansion or corolla opening compared with untreated controls (Supplementary Table S2). These results suggest that impaired corolla opening in *RUBY*-overexpressing lines is unlikely to be explained by GA limitation alone and may involve additional regulatory networks that remain to be elucidated.

A strong inverse correlation was observed between the degree of flower opening and red pigmentation intensity; flowers with more intense red coloration opened less fully. Fully red flowers consistently had a reduced corolla aperture, often hindering the observation of stigma and anthers.

To further validate that the observed phenotypic changes were specifically due to *RUBY* expression rather than the transformation process or vector effects, we also compared *RUBY* transgenic plants with empty vector control plants (pKI1.1R, Addgene Plasmid #85808). These empty vector controls, which underwent the same *Agrobacterium*-mediated transformation and regeneration procedure as the *RUBY* lines, exhibited phenotypes indistinguishable from wild-type (WT) plants (Supplementary Figure S3), confirming that the altered petal expansion and flower opening in *RUBY* lines are attributable to *RUBY* expression.

A qRT-PCR analysis further revealed that *RUBY* gene expression was higher in floral organs with deeper red pigmentation and reduced flower opening. Moreover, key gibberellin (GA) biosynthesis genes, *PhGA20ox1* and *PhGA3ox1*, were consistently downregulated in *RUBY* lines, implying that suppressed GA synthesis may limit petal expansion. The flowering regulator *PhNF-YC2* and bZIP transcription factor *PhOBF1*, both involved in GA synthesis and floral senescence, were also repressed (Figure 6B–F). Collectively, these findings suggest that exogenous *RUBY*-mediated betalain synthesis perturbs hormone signaling and transcriptional regulation, ultimately reducing flower opening in *Petunia*.

## 3. Discussion

### 3.1. A High Cytokinin Concentration Enhances Petunia Regeneration via Meristematic Activation

We observed efficient callus induction and shoot regeneration using a BAP concentration in the regeneration medium, consistent with the role of cytokinins in promoting cell division and meristem maintenance [16,17]. Cytokinins activate WUSCHEL and other stem cell regulatory networks, preserving pluripotency during in vitro culture. The enhanced regeneration observed under a high cytokinin/auxin ratio aligns with established hormonal crosstalk models for the control of organogenesis [18]. In addition, cytokinins have been implicated in the regulation of cell cycle progression and chloroplast development, which are critical for tissue regeneration [19,20]. Because regeneration efficiency is often a limiting step in transformation workflows, optimizing the hormone balance is essential for achieving scalable genetic engineering in petunia.

### 3.2. RUBY as a Non-Invasive, Robust Visual Marker for Plant Transformation

The successful application of *RUBY* as a visual marker corroborates recent advances in metabolic reporter genes that circumvent the limitations of *GFP* and *GUS* systems [21,22,23]. *RUBY*’s stable red pigmentation, deriving from the heterologous activation of betalain biosynthesis, facilitates early and continuous detection of transformed tissues without exogenous substrates or specialized imaging equipment. Chimerism in T_0_ plants likely reflects multicellular origins and partial T-DNA incorporation, a phenomenon widely reported in *Agrobacterium*-mediated transformation [24,25]. Future approaches, such as single-cell lineage tracking and optimization of explant tissue origin, may mitigate mosaicism and improve transgene uniformity.

Although reduced petal expansion and incomplete flower opening were observed in *RUBY* transgenic lines, vegetative growth and fertility were not significantly affected, and the plants produced viable seeds. Microscopic examination revealed that protoplasts isolated from the leaves of both *RUBY*-transformed and WT plants were intact, turgid, and morphologically normal, with no evidence of plasmolysis or structural abnormalities. These findings suggest that *RUBY* expression does not negatively affect cell viability or structural stability. Reduced expression of *PhGA20ox1* and *PhGA3ox1* was observed in transgenic lines with high *RUBY* expression. These findings suggest that careful consideration may be required when employing *RUBY* in studies directly involving hormonal regulation or floral organ development. Nevertheless, as a visual marker for identifying transgenic events, *RUBY* remains a practical and efficient reporter system.

In protoplast transient transformation systems, *GFP* plasmids are commonly employed as reporter genes owing to their small size (5667 bp), stable expression, and ease of detection [26]. To evaluate the potential of *RUBY* in such systems, we replaced *GFP* with the *35S:RUBY* plasmid as a reporter. The transformation efficiency in our assays was extremely low, and no visible pigment accumulation could be reliably detected (Supplementary Figure S2). This could be attributed to the substantially larger total size of the *35S:RUBY* plasmid (14,335 bp) than that of *GFP*, as an increased vector size has been shown to significantly reduce DNA uptake and nuclear import efficiency [27,28]. Therefore, while *RUBY* serves as an efficient, stable, and non-destructive visual marker for whole-plant transformation, it is unsuitable for use as a reporter in protoplast transient transformation assays. This finding underscores the need to consider transformation efficiency, vector size, and expression kinetics when selecting an appropriate visual reporter system [29].

### 3.3. Fine-Tuning Infection and Pre-Culture Conditions Is Critical to Transformation Success

Our results highlight the dual-edge nature of *Agrobacterium* infection time and pre-culture duration on transformation outcomes. Excessive infection fosters bacterial overgrowth, leading to tissue necrosis and lowered transformation rates. Conversely, insufficient infection yields poor T-DNA delivery. The optimal infection period of 20 min balances these factors, in agreement with transformation protocols optimized for tomato (*Solanum lycopersicum* L.) and cotton (*Gossypium hirsutum* L.) [30,31,32]. The beneficial effect of a one-day pre-culture aligns with the theory that moderate wounding and cell cycle synchronization enhance competence for T-DNA uptake. Overextended pre-culture likely triggers defense pathways or programmed cell death, reducing transformation efficiency [33,34,35]. These findings underscore the necessity of species- and genotype-specific protocol refinement.

### 3.4. RUBY Expression Is Correlated with Betalain Accumulation and Pigment Intensity

We found a positive correlation between the *RUBY* transcript level, visible red pigment intensity, and betalain content in transgenic plants. As a synthetic transcription factor in the betalain biosynthetic pathway, *RUBY* can activate betalain synthesis in non-Caryophyllales species, consistent with previous results [8,36,37]. Although we discovered that water is also an effective solvent for betalain extraction, a 40% methanol solution can yield betalain with higher purity and concentration. This finding corroborates previous studies indicating that solvents of intermediate polarity represent the optimal choice for betalain extraction [38,39]. Collectively, these findings validate the functional utility of the *RUBY* system and underscore its potential as a versatile metabolic engineering tool for natural pigment biosynthesis and the development of value-added crop traits. The *RUBY* reporter gene, integrated into a CRISPR/Cas9 vector, has also been employed by Chen and his team [40] to rapidly identify a transgene-free *Gmwaxy* mutant in the T_1_ generation. Additionally, the introduction of a *RUBY* expression construct into white cotton enabled the generation of a novel pink fiber phenotype without the use of exogenous dyes, while in soybean (*Glycine max*), it facilitated the rapid identification of transgene-free mutants [40,41]. Further refinement of *RUBY* expression and pathway flux may enhance betalain accumulation and extend the applicability of this system to a broader range of plant species.

### 3.5. RUBY-Mediated Pigmentation and Flower Opening in Petunia

A novel and intriguing finding is the inverse relationship between betalain accumulation and flower opening in petunia, accompanied by changes in hormone-related and transcriptional gene expression. In *Solanaceae*, flower opening is largely driven by petal cell expansion, a process promoted by gibberellin (GA) signaling [42,43]. The observed downregulation of *PhGA20ox1* and *PhGA3ox1* in *RUBY* transgenics indicates an association between betalain accumulation and altered GA-related gene expression. In *Arabidopsis thaliana*, disruption of GA homeostasis has been shown to affect floral development [44]. In addition, the reduced expression of *PhNF-YC2*, a photoperiod-related flowering regulator [45], and *PhOBF1*, a bZIP transcription factor known to enhance GA synthesis and antagonize ethylene-mediated senescence [46,47], suggests the possibility of broader hormonal alterations in *RUBY* plants. Such cross-pathway interference may arise from metabolic competition; betalain biosynthesis originates from tyrosine, a branch point of the shikimate pathway, which is also upstream of multiple hormone-related metabolites [11]. In non-Caryophyllales species, such as petunia, the artificial introduction of this pathway could disrupt metabolic homeostasis, leading to pleiotropic effects on developmental gene networks.

Previous studies have shown that betalain biosynthetic gene expression is developmentally regulated during floral development and anthesis in betalain-producing species, with key genes such as *DOD* and *CYP76AD* displaying stage-dependent expression patterns during flower maturation [48]. In this context, strong ectopic *RUBY* expression may intersect with endogenous floral regulatory programs. Notably, the genes examined in the present study (*PhGA20ox1*, *PhGA3ox1*, *PhNF-YC2*, and *PhOBF1*) are associated with hormone biosynthesis and transcriptional regulatory networks. These genes were selected for expression analysis because they represent key regulators of gibberellin biosynthesis and floral developmental signaling pathways known to influence petal growth, flower maturation, and corolla opening in ornamental plants. In contrast, the canonical betalain biosynthetic genes reported in previous studies (e.g., *DOD*, *CYP76AD*, *B5GT*) belong primarily to pigment metabolic pathways. Therefore, the transcriptional changes observed in *RUBY* transgenic lines likely represent downstream regulatory responses rather than direct regulation by betalain biosynthetic enzymes. Furthermore, transcription factors known to regulate betalain biosynthesis, including *MYB*, *bHLH*, *WRKY*, and *SPL*, are also widely involved in broader developmental and hormonal regulatory networks, suggesting that pigment biosynthesis and floral developmental programs may share overlapping regulatory components [49]. Gibberellins are well-established regulators of floral organ growth and petal expansion in ornamental plants, and exogenous GA_3_ application has been reported to promote bud development and flower opening in species such as petunia, gerbera, and rose [50,51]. Consistent with this, the downregulation of *PhGA20ox1* and *PhGA3ox1* in *RUBY* transgenic lines may be associated with reduced petal expansion and incomplete corolla opening. However, exogenous GA_3_ treatment did not restore corolla expansion in the present study, indicating that the impaired flower-opening phenotype cannot be explained solely by reduced GA biosynthesis. Instead, flower opening likely involves complex hormonal interactions and developmental regulatory networks, and the phenotype observed in *RUBY* lines may arise from broader transcriptional or metabolic perturbations associated with high-level betalain accumulation.

However, the direct causal factors underlying *RUBY*-induced abnormal flower opening remain to be fully elucidated. While our study provides evidence for the influence of certain hormone pathway genes and transcription factors, the precise molecular mechanism directly linking betalain accumulation to petal expansion and flower opening warrants further in-depth investigation.

From an applied perspective, the suppression of floral opening may reduce pollinator visibility, potentially limiting reproductive success in natural conditions but offering ornamental breeding opportunities for novel, compact floral phenotypes with enhanced pigment intensity. Although exogenous GA_3_ application did not restore corolla opening in our study, future work should quantify endogenous GA levels in *RUBY* petals and employ transcriptomic–metabolomic integration to elucidate potential signaling cross-talk between betalain metabolism and floral morphogenesis.

## 4. Materials and Methods

### 4.1. Plant Material and Culture Conditions

*Petunia***×***hybrida* cv. ‘Madness Midnight’ seeds were obtained from Panamseed Company Pvt. Ltd. (Daejeon, Republic of Korea). The seeds were surface-sterilized in a 2% NaClO solution (Sigma-Aldrich, St. Louis, MO, USA) for 15 min, followed by five rinses with sterile distilled water. Sterilized seeds were then transferred to half-strength Murashige and Skoog (½ MS) solid medium (Duchefa Biochemie, Haarlem, The Netherlands) supplemented with 3% (*w*/*v*) sucrose and 0.8% (*w*/*v*) agar, with the pH adjusted to 5.8. The seeds were incubated in a controlled culture chamber at 25 °C, with a light intensity of 98–117 μmol·m^−2^·s^−1^ and a photoperiod of 16 h light/8 h dark. Transgenic and WT control plants were maintained under identical environmental conditions to ensure experimental consistency.

### 4.2. Plasmid Vector

The plasmid *35S:RUBY* (#160,908) was obtained from Addgene (www.addgene.org). It was originally deposited by Prof. Yunde Zhao, University of California (San Diego, CA, USA) [8]. The empty vector plasmid pKI1.1R (Addgene Plasmid #85808), a Cas9 construct lacking any sgRNA sequence, was used as a transformation control. All plasmids were introduced into *Agrobacterium tumefaciens* strain GV3101 using the freeze–thaw method for plant genetic transformation.

### 4.3. Agrobacterium-Mediated 35S:RUBY Transformation

The fifth true leaf of 3- to 4-week-old petunia plants was excised, and leaf disc explants (0.25 cm × 0.5 cm) were prepared. The petunia leaf disc explants were precultured for 0–2 days on preculture medium containing 2 mg/L BAP + 0.5 mg/L NAA under dark conditions.

Recombinant *Agrobacterium* cultures were subcultured in 20 mL of LB medium at 28 °C with continuous shaking (200 rpm) overnight. Cells were harvested at OD_600_ = 0.5–0.6 by centrifugation at 5000 rpm for 8 min and resuspended in 20 mL of MS liquid medium supplemented with 100 µM acetosyringone. The suspension was incubated for 5–30 min under the same conditions to activate *vir* gene expression before infection.

The precultured explants were infected with the recombinant *Agrobacterium* suspension for 5–30 min in the dark under continuous shaking. Following infection, the explants were gently blotted dry on sterile filter paper and transferred to a co-cultivation medium containing 100 µM acetosyringone, 1 mg/L BAP, and 0.5 mg/L NAA for 2 days. After the co-cultivation period, the explants were thoroughly washed with sterile water and ½ MS liquid medium supplemented with 500 mg/L cefotaxime to eliminate excess bacteria. The cleaned explants were then transferred to two types of selection medium. Medium A, which included 1 mg/L BAP + 0.5 mg/L IBA [52], and Medium B, which consisted of 2 mg/L BAP + 0.5 mg/L NAA [53]. Both media contained 10 mg/L hygromycin B, and the explants were cultured under light conditions. Callus induction and subsequent shoot regeneration were monitored regularly.

### 4.4. Molecular Confirmation of Transgenic Plants

Total genomic DNA was extracted from 100 mg of putatively transformed plant leaves. The extraction was carried out using the DNeasy Plant Mini Kit (QIAGEN, Hilden, Germany) after tissue grinding in liquid nitrogen. To confirm the presence of the *RUBY* gene in T_0_ and T_1_ *RUBY* transgenic plants, PCR was performed using *RUBY* gene-specific primers. The amplified PCR products were then separated by electrophoresis on a 1.5% agarose gel. Banding patterns were subsequently visualized and documented.

### 4.5. RNA Extraction and qRT—PCR Analysis

Petunia tissues were pulverized using liquid nitrogen. Subsequently, total RNA was extracted from 100 mg of leaves using the GeneAll Hybrid RNA Purification Kit (GeneAll Biotechnology, Daejeon, Republic of Korea). Using approximately 2 μg of total RNA as a template, complementary DNA (cDNA) was synthesized by means of the PrimeScript RT Reagent Kit (Takara Korea Biomedical, Seoul, Republic of Korea). Gene-specific forward and reverse oligonucleotide primers for the *RUBY* and 26S ribosomal RNA were designed using PrimerPremier5 software for qRT-PCR (Supplementary Table S1). Based on three biological replicates and three technical replicates, the relative expression levels of the target gene transcripts were assessed by applying the 2^−ΔΔCt^ method.

### 4.6. Betalain Extraction and Quantification

The protocol was adjusted according to previously described methods [38]. Five hundred milligrams (fresh weight) of petunia leaves were ground into powder in liquid nitrogen. Subsequently, the powder was suspended in methanol and extracted for 20 min. The mixture was centrifuged at 10,000 rpm and 4 °C for 10 min, and the supernatant containing betalain was collected. After adjusting the pH to 3–5 with HCl, the sample was centrifuged again at 10,000 rpm and 4 °C for 10 min. Four milliliters of trichloromethane (TCM) were added to 1 mL of the crude sample for extraction and separation. The mixture was vortexed for 10 min and allowed to stand for 5 min for phase separation. The upper phase was collected by centrifugation at 10,000 rpm and 4 °C for 10 min. Finally, the betalain concentration was determined spectrophotometrically.

The concentrations of betacyanin (BE) and betaxanthin (IE) were estimated using the following formula [54,55]:

$$\mathrm{Betalain}(\mathrm{mg}/100\;\mathrm{mL})=\frac{(A_{\max}-A_{600\;\mathrm{nm}})\times \mathrm{Dilution}\;\mathrm{Factor}\times \mathrm{MW}\times 100}{(Ɛ\times 1)}$$

Betalain concentration(mg/100 mL)=BE+IE

Betacyanin: A_max_  =  532 nm, MW  =  550 g/mol, and ε  =  60,000 L mol^−1^ cm^−1^ (betanin 153 equivalent (BE)).

Betaxanthin: A_max_  =  482 nm, MW  =  308 g/mol, and ε  =  48,000 L mol^−1^ cm^−1^ (indicaxanthin equivalent (IE)).

### 4.7. Exogenous GA_3_ Treatment

Exogenous GA_3_ treatment was performed on two-month-old transgenic plants (*RUBY*-1). GA_3_ was dissolved in a small volume of ethanol and diluted to final concentrations of 100 μM with distilled water containing 0.01% Tween-20. The solution was sprayed onto the plants once daily for three consecutive days. One week later, corolla opening was evaluated and compared with that of the untreated control group (Supplementary Table S2).

### 4.8. Statistical Analysis

Unless otherwise specified, all experiments were carried out with a minimum of three independent biological replicates. Quantitative data are presented in the form of means ± standard deviations (SD).

Prior to analysis of variance (ANOVA) test, normality was assessed using the Shapiro–Wilk test, and homogeneity of variances was evaluated using Levene’s test. For comparisons between two groups, statistical significance was evaluated using an unpaired two-tailed Student’s *t*-test. For experiments involving more than two groups, one-way ANOVA was conducted followed by Tukey’s post hoc test for multiple comparisons. When transgenic lines were compared specifically with the WT control, Dunnett’s multiple comparisons test was applied. A *p*-value of less than 0.05 was regarded as statistically significant. Statistical analyses were performed using IBM SPSS Statistics 20. All figures and graphs were generated using Adobe Photoshop 2025 and GraphPad Prism 9.5.

## 5. Conclusions

This study establishes the *RUBY* reporter gene as a powerful and convenient visual marker for *Agrobacterium*-mediated transformation in *Petunia* × *hybrida*, facilitating rapid, non-invasive identification of transgenic tissues throughout regeneration. Optimizing key factors—including a high cytokinin concentration (2 mg/L BAP), one-day pre-culture, and a 20 min infection period—enhanced transformation efficiency significantly, reaching over 70% in favorable conditions. The strong correlations between *RUBY* expression, betalain accumulation, and visible red pigmentation validate *RUBY* as a reliable dual-function marker for both transformation screening and metabolic engineering.

Importantly, our data reveal that heterologous expression of *RUBY* and consequent betalain biosynthesis in petunia perturb endogenous hormone biosynthesis and gene regulatory networks, leading to reduced flower opening. This provides novel insights into the metabolic–hormonal crosstalk arising from pathway engineering in non-native species. Such pleiotropic effects pose challenges but also opportunities to develop new ornamental traits combining intense pigmentation with altered floral architectures.

Future studies should leverage multi-omics integration, hormone quantification, and genetic manipulation to further dissect the regulatory interactions between introduced metabolic pathways and host developmental programs. This knowledge will advance the precision engineering of complex traits in ornamental and crop plants, expanding the utility of *RUBY* and similar metabolic reporters in plant biotechnology.

## Figures and Tables

**Table 1:** Segregation of phenotypes in different lines of the *35S:RUBY* transgenic *Petunia* T1 generation.

			Surviving Seedings (%)				
*35S: RUBY* Transgenic lines (T1)	Total Number of Seedings	Number of Germination	Fully Green (%)	Semi-Red (%)	Fully Red (%)	Dead Seedings (%)	The Ratio of Surviving Seedings (%)
***RUBY*** **-1**	**88**	**80**	**31.25%**	**62.50%**	**6.25%**	**9.09%**	**90.91%**
***RUBY*** **-2**	**57**	**40**	**15.00%**	**60%**	**25%**	**29.82%**	**70.18%**
***RUBY*** **-9**	**130**	**110**	**25.45%**	**53.64%**	**20.91%**	**15.38%**	**84.62%**

Note: The segregation data were summarized descriptively to demonstrate the heritability of the *RUBY*-associated pigmentation phenotype. A formal χ^2^ test and transgene copy number analysis were not performed in this study.

**Table 2:** Petal phenotypes of WT and T0 *RUBY* lines.

Line	Total Petals	Closed Flowers (Fully Red)	Partially Open Flowers (Purple-Red Stripes)	Fully Open Flowers (Fully Purple)	Mean Petal Diameter (cm) ± SD	Mean Petal Diameter *p*-Value (vs. W)T
WT	18	0	0	18	5.24 ± 0.77	-
*RUBY*-1	16	8	5	3	3.80 ± 1.02	<0.000 ***
*RUBY*-2	13	8	3	2	3.61 ± 1.04	<0.000 ***
*RUBY*-3	17	1	6	10	5.11 ± 0.79	1.000 ns
*RUBY*-4	11	0	2	9	5.45 ± 0.58	1.000 ns
*RUBY*-5	9	0	3	6	5.21 ± 0.69	1.000 ns
*RUBY*-6	19	1	4	14	5.05 ± 0.83	0.999 ns
*RUBY*-7	17	2	4	11	4.92 ± 1.17	0.948 ns
*RUBY*-8	10	1	2	7	4.83 ± 1.24	0.913 ns
*RUBY*-9	16	14	2	0	3.23 ± 0.79	<0.000 ***
*RUBY*-10	12	2	6	4	4.62 ± 1.04	0.435 ns
*RUBY*-11	11	0	3	8	5.18 ± 0.60	1.000 ns
*RUBY*-12	18	1	5	12	5.28 ± 0.84	1.000 ns

Data are presented as mean ± SD. Statistical differences were analyzed using one-way ANOVA followed by Dunnett’s multiple comparisons test against the WT control. *** *p* < 0.001; ns, not significant.

## Data Availability

The original contributions presented in this study are included in the article/Supplementary Material. The datasets generated and/or analyzed during the current study are available from the corresponding author on reasonable request. The data can be publicly available.

## Supplementary Materials

The following supporting information can be downloaded at: https://www.mdpi.com/article/10.3390/plants15060886/s1.
