# Supplementary material for: RUBY-Mediated Visual Selection Facilitates Transgenic Screening and Red Floral Pigmentation in Petunia × hybrida
Source: Plants (Basel). 2026 Mar 12;15(6):886. doi: 10.3390/plants15060886 (PMC13029396; doi:10.3390/plants15060886)
Supplement: Supplementary file 1 [file plants-15-00886-s001.zip › plants-4171026-supplementary.pdf]

Supplementary Table S1. Primers used for qRT-PCR analysis.

**Gene**	**Forward primer (5′-3′)**	**Reverse primer (5′-3′)**	**Product size**
RUBY	AACAGCATCCTTGAGTCTCTTCG	TTCTCTTTGGAGATCTCGCCTTC	152 bp
26S ribosomal RNA	AGCTCGTTTGATTCTGATTTCCAG	GATAGGAAGAGCCGACATCGAAGG	185 bp
PhOBF1	AACATGACTACAACACAATATGC	GATAGAACATATCAGCTGAAGCC	268 bp
PhGA20ox1	TGCTTGCATAGAGCAGTTGTAAA	GAGTTCTCTTGTCAGCCCTGTAA	196 bp
PhGA3ox1	TCACTAGGAATAACCAAGGACGA	ACTACCGCTCGATGTAACACACT	299 bp
qPhNF-YC2	TGAGCAGTGCCTCTTGTTACC	TGAGGTAATTTGATCGCGAGGT	199 bp

Supplementary Table S2. Comparison of mean petal diameter between *RUBY*-1 plants treated with water and exogenous GA_3.

Line	Mean Petal Diameter (cm) ± SD	*P*-value
RUBY-1+ water	3.03 ± 0.68	
RUBY-1+GA_3 (100μM)	3.15 ± 0.84	0.978 ns

Note: Data are presented as mean ± SD (n = 5 biological replicates per group). Statistical analysis was performed using an unpaired two-tailed Student's t-test after confirming homogeneity of variance. No significant difference was observed between groups (p = 0.978).

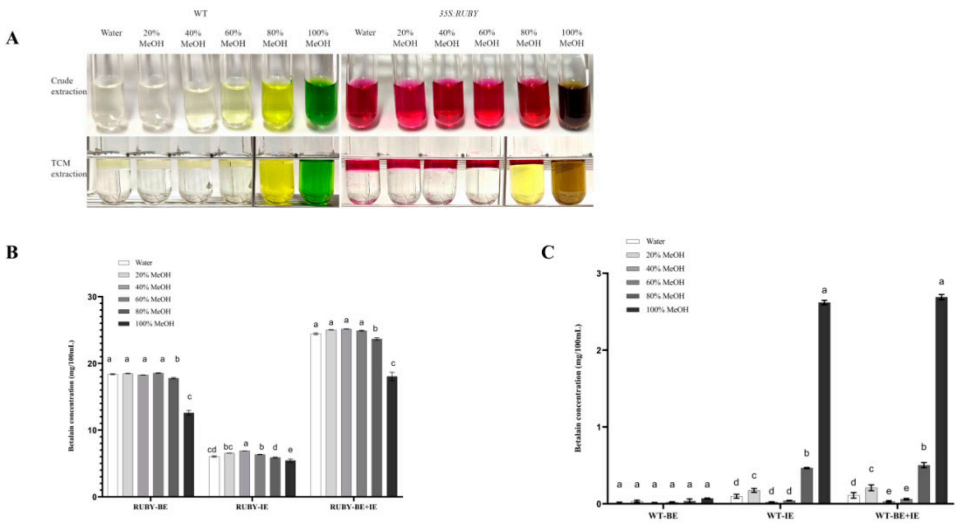

Supplementary Figure S1. Betalain accumulation and quantification in wild-type and transgenic petunia under different extraction conditions. (A) Crude and trichloroacetic acid–methanol (TCM) extracts of tissues from wild-type (WT) and *RUBY* transgenic plants using water and different concentrations of methanol (MeOH). WT samples remained colorless or yellowish, while transgenic tissues showed strong red pigmentation due to betalain accumulation, especially under high MeOH concentrations. (B–C) Quantification of betalain content by spectrophotometry. Absorbance values were measured at 532 nm, 482 nm and 600 nm for crude extracts and TCM extracts from WT and *RUBY* transgenic plants. Betacyanin and betaxanthin concentrations were calculated based on molar extinction coefficients and molecular weights, following a modified protocol from Liu et al. (2024). For each biological replicate, plant tissues were collected from independent plants. Absorbance was measured in triplicate for each sample, and the mean value was used for statistical analysis. Data are presented as mean ± SD (n = 3 biological replicates). Statistical differences among treatments were analyzed by one-way ANOVA followed by Tukey's multiple comparison test ($P < 0.05$).

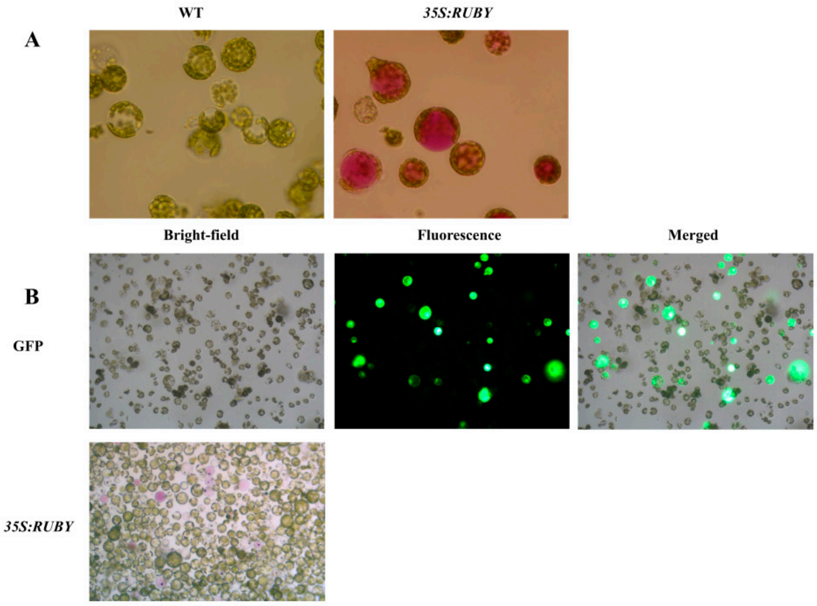

Supplementary Figure S2. Protoplast extraction and transient expression in petunia. (A) Phenotypes of protoplasts isolated from WT and *35S:RUBY* transgenic petunia leaves. Red pigmentation is visible in *RUBY* plant protoplasts, while protoplasts from WT leaves appear greenish. (B) Transient expression of GFP and *35S:RUBY* plasmid in petunia protoplasts. Left: Bright-field channel; Middle: Fluorescence channel; Right: Merged image showing GFP fluorescence in successfully transformed cells.

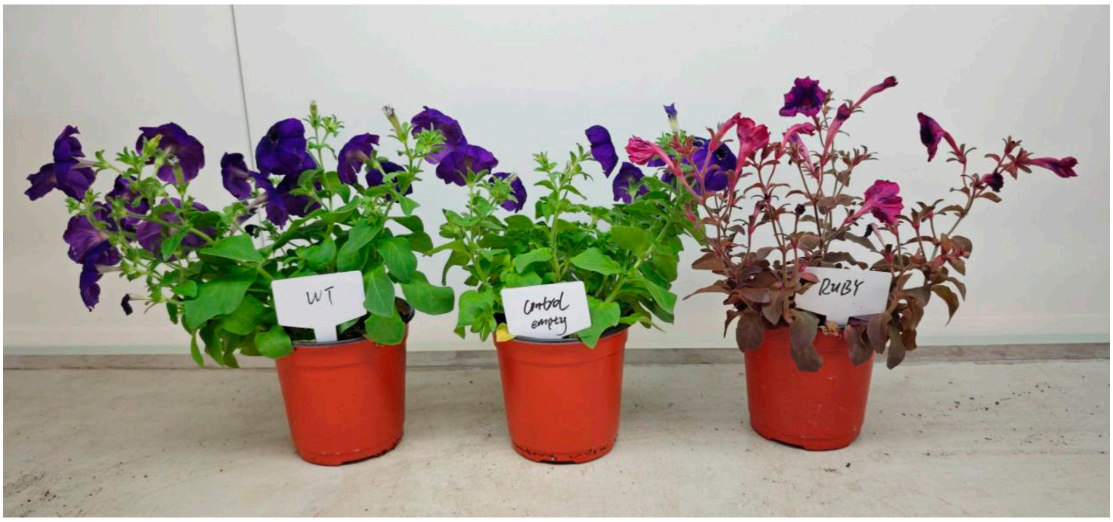

Supplementary Figure S3. Phenotypic comparison of wild-type (WT), empty vector control (pKI1.1R, Addgene Plasmid #85808; without sgRNA), and *RUBY* transgenic plants. Empty vector controls exhibit similar phenotypes to WT, while *RUBY* plants show the expected traits.
